# Radiofrequency applicator concepts for thermal magnetic resonance of brain tumors at 297 MHz (7.0 Tesla)

**DOI:** 10.1080/02656736.2020.1761462

**Published:** 2020-06-02

**Authors:** Eva Oberacker, Andre Kuehne, Celal Oezerdem, Jacek Nadobny, Mirko Weihrauch, Marcus Beck, Sebastian Zschaeck, Cecilia Diesch, Thomas Wilhelm Eigentler, Helmar Waiczies, Pirus Ghadjar, Peter Wust, Lukas Winter, Thoralf Niendorf

**Affiliations:** a Berlin Ultrahigh Field Facility (B.U.F.F.), Max-Delbrück-Center for Molecular Medicine in the Helmholtz Association, Berlin, Germany; b Department of Physics, Faculty of Mathematics and Natural Sciences, Humboldt-Universität zu Berlin, Berlin, Germany; c MRI.TOOLS GmbH, Berlin, Germany; d Experimental and Clinical Research Center (ECRC), Joint Cooperation Between the Charité Medical Faculty and the Max-Delbrück-Center for Molecular Medicine in the Helmholtz Association, Berlin, Germany; e Clinic for Radiation Oncology, Charité Universitätsmedizin, Berlin, Germany; f Berlin Institute of Health (BIH), Berlin, Germany; g Chair of Medical Engineering, Technische Universität Berlin, Berlin, Germany; h Physikalisch Technische Bundesanstalt, Braunschweig, Germany

**Keywords:** RF hyperthermia, thermal magnetic resonance, hyperthermia treatment planning, glioblastoma multiforme, magnetic resonance imaging, magnetic resonance thermometry

## Abstract

**Purpose:**

Thermal intervention is a potent sensitizer of cells to chemo- and radiotherapy in cancer treatment. Glioblastoma multiforme (GBM) is a potential clinical target, given the cancer’s aggressive nature and resistance to current treatment options. The annular phased array (APA) technique employing electromagnetic waves in the radiofrequency (RF) range allows for localized temperature increase in deep seated target volumes (TVs). Reports on clinical applications of the APA technique in the brain are still missing. Ultrahigh field magnetic resonance (MR) employs higher frequencies than conventional MR and has potential to provide focal temperature manipulation, high resolution imaging and noninvasive temperature monitoring using an integrated RF applicator (ThermalMR). This work examines the applicability of RF applicator concepts for ThermalMR of brain tumors at 297 MHz (7.0 Tesla).

**Methods:**

Electromagnetic field (EMF) simulations are performed for clinically realistic data based on GBM patients. Two algorithms are used for specific RF energy absorption rate based thermal intervention planning for small and large TVs in the brain, aiming at maximum RF power deposition or RF power uniformity in the TV for 10 RF applicator designs.

**Results:**

For both TVs , the power optimization outperformed the uniformity optimization. The best results for the small TV are obtained for the 16 element interleaved RF applicator using an elliptical antenna arrangement with water bolus. The two row elliptical RF applicator yielded the best result for the large TV.

**Discussion:**

This work investigates the capacity of ThermalMR to achieve targeted thermal interventions in model systems resembling human brain tissue and brain tumors.

## Introduction

Glioblastoma multiforme (GBM) is the most frequent and aggressive malignant brain tumor with the least chance of long-term survival, in spite of multimodal therapeutic approaches that include surgery, radiotherapy and chemotherapy [1]. Local thermal therapy is a potent sensitizer of several cancer cell types to chemo- and radiotherapy [2–5] and significantly improves survival [5]. In case of GBM, adding thermal therapy to standard treatment could improve prognosis [1,5]. A randomized trial showed the principal effectiveness of brachytherapy and adjunct interstitial hyperthermia, prolonging median survival [6]. The disproportional effort and burden of this approach has prevented broader clinical application and constitutes the rationale for a noninvasive approach for thermal therapy. GBM treatment could also benefit from manipulation of the blood brain barrier enhancing its permeability and thus targeted drug delivery to the tumor location [7–9].

Limitations of energy delivery restrict the use of conventional thermal therapy approaches in the brain. Capacitive approaches were already clinically applied [10]. Their ability to achieve high SAR in deep tissue and to focus to a well-defined target region is limited [11,12]. MR-guided focused ultrasound (MRgFUS) permits noninvasive thermo-ablation of deep seated brain tumors [13]. This approach provides unmatched focal quality [14], although excessive treatment duration often results in incomplete treatment of the clinical target volumes (TVs) [15].

Radiative annular phased arrays (APAs) of antennae operating in the radiofrequency (RF) range enable localized temperature intervention in deep-seated TVs in the pelvis and abdomen [2], where the commonly applied frequencies of *f* = 70–130 MHz result in wavelengths *λ* between 50 and 25 cm and thus the minimum size of the heated volume is as large as about 10 cm (about one-third of the wavelength) [16,17]. Employing a higher frequency of *f* = 434 MHz affords smaller TVs in the treatment of head and neck cancers [18]. Numerical simulations demonstrated that moving to frequencies of up to 1 GHz and a potential combination thereof enables better control over the distribution of the delivered RF energy to the target [19–22].

A plethora of reports highlights the need and growing clinical interest for RF-based thermal therapy in the brain [19,21–24]. However, reports on clinical applications of the APA technology are still missing due to the lack of a noninvasive method to manipulate brain tissue temperature while concomitantly characterizing its outcome *in vivo*, which is a pivotal prerequisite when targeting the brain. Temperature probes provide accurate readings but are constrained to point-wise measurements. Their placement is invasive and might not be feasible depending on the size and location of the TV.

Magnetic resonance imaging (MRI) provides anatomic reference, facilitates functional contrast and supports *noninvasive* temperature mapping and is therefore of great interest but has still limited accessibility for clinical users [12,25]. Current implementations use separate RF antenna systems for RF hyperthermia and MRI [12]. For the latter, the MR scanner’s body RF coil is employed. This approach constrains MR thermometry (MRTh) to a low signal-to-noise ratio, which limits the spatial resolution and accuracy of temperature maps [26,27]. Further constraints apply to the design of the RF heating device: the RF antenna system for hyperthermia must be efficiently decoupled from the MR imaging system to avoid RF induced artifacts in the MRTh data as well as perturbations in the RF transmission during treatment [2,28].

Thermal magnetic resonance (ThermalMR) has the unique potential to circumvent these limitations by providing temperature intervention, proton (^1^H) MRI for anatomic and functional imaging and temperature mapping (MRTh) in an integrated RF applicator. Ultrahigh field MR (UHF-MR) at *B*_0_≥7.0 Tesla (*f* ≥ 297 MHz) enables high focusing of RF fields, provides thermal dose delivery for hyperthermia in relatively large tumor areas and affords enhanced spatial resolution for MRI and MRTh [20,23,27,29,30]. By enabling treatment and therapy monitoring on the same RF antenna hardware, the risk of interferences between treatment and therapy monitoring is eliminated.

Recognizing the opportunities of adding a thermal intervention dimension to a UHF-MR device, this work examines RF applicator concepts tailored for simultaneous RF heating and UHF-MR to combine diagnostic MRI, RF hyperthermia treatment and real-time therapy control with MRTh. En route to RF-induced hyperthermia treatment of glioblastoma in the human brain, this work focuses on electromagnetic field (EMF) simulations that incorporate models based on clinical data obtained from GBM patients and presents two algorithms for specific RF energy absorption rate (SAR)-based hyperthermia treatment planning (HTP) for small and large TVs in the brain.

## Materials and methods

### Radiofrequency antenna array concepts

**Figure 1.**
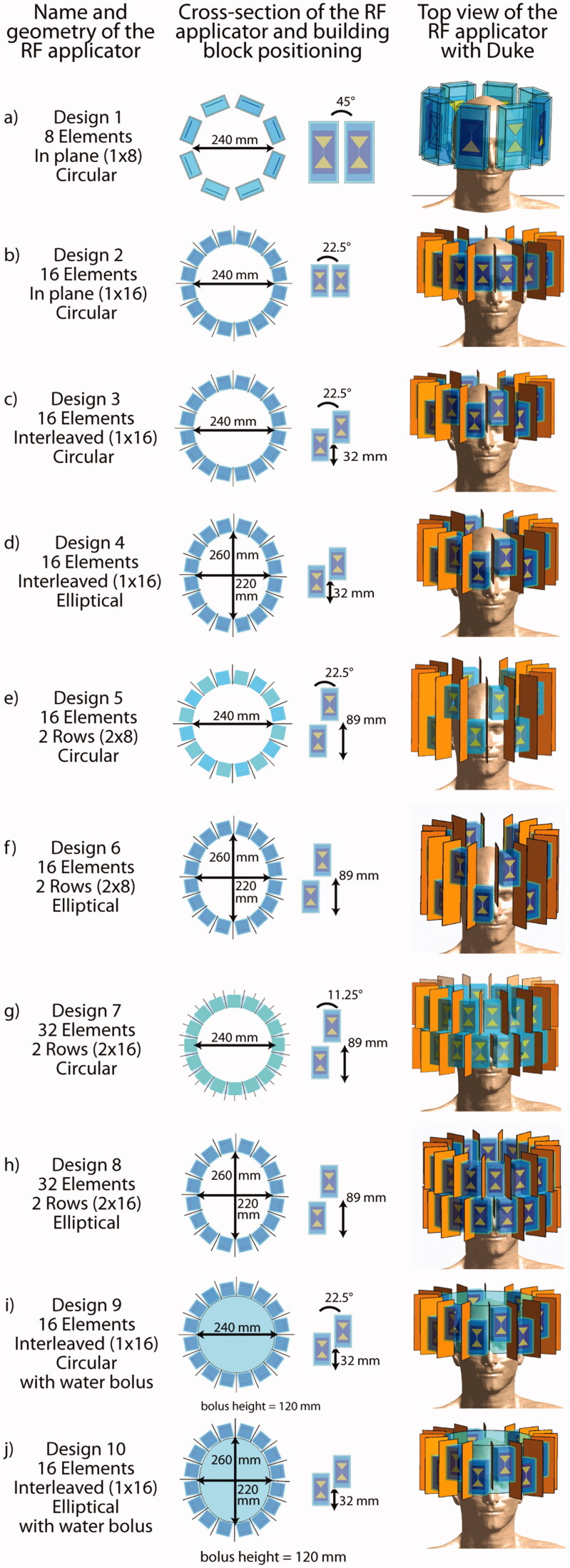
Overview over all 10 RF applicator designs investigated in this work. In the center column, cross-sectional view of the RF arrays depicts the arrangement of the building blocks around the head and details about their position with respect to each other. The rightmost column shows the designs and their positioning for the small tumor model.

Ten RF arrays comprising 8, 16 or 32 antenna building blocks (Figure 1) were modeled. Each building block consists of a bow tie dipole antenna [23,31] submerged in a high permittivity medium enclosed in a polymethylmethacrylate (PMMA) box (wall thickness = 3 mm).

*Design 1* (Figure 1(a)) provides a reference [22] and consists of eight building blocks (size: (35 × 70 × 150) mm³) positioned azimuthally (inner diameter = 24 cm) around the human head. D_2_O (*ε*_r_ ≈ 80) was used as high permittivity medium for antenna size reduction [32].

For *Designs 2–6*, the number of transmit channels was increased to 16 versus the reference design, which potentially improves in-plane RF steering capabilities and the ratio between deep seated and superficial SAR [20]. In order to allow for the increased number of channels, while keeping the overall dimensions of the applicator comparable, a higher permittivity dielectric (*ε*_r_ ≈ 200) was employed, which reduced the building block size to (40 × 40 × 80) mm³.

*Design 2* (Figure 1(b)) comprises 16 resonators arranged in a circular array in plane.

Starting with *Design 3* (Figure 1(c)), an interleaved arrangement of building blocks along the head-feet-direction (*z*-direction) was introduced in order to sharpen and move the focus along the third dimension. *Designs 3 and 4* use an offset of 32 mm between adjacent building blocks, which represents half the length of the dipole.

*Design 4* (Figure 1(d)) is an elliptical variant of Design 3 to reduce the proximity of the elements to the front and the back of the head and to mitigate formation of local SAR maxima. The eccentricity of the ellipse (*a*_1_=220 mm, *a*_2_=260 mm) was chosen to reproduce the circumference of the circular arrays.

To enhance brain coverage, the offset of the building blocks in head-feet-direction was set to the length of the building block (89 mm) for *Designs 5–8*, forming two separate rows.

*Design 5* (Figure 1(e)) is a circular two row arrangement with eight elements each.

*Design 6* (Figure 1(f)) is an elliptical variant of Design 5 with improved longitudinal coverage over Design 4.

For *Designs 7 and 8*, 32 building block elements were used. The circular *Design 7* (Figure 1(g)) consists of two rows, each equipped with 16 elements.

*Design 8* (Figure 1(h)) is an elliptical variant of Design 7 with an enhanced conformity to the shape of the head. Note that for Designs 1–8 an air gap is assumed between antennae and head.

Building block arrangements in *Designs 9 and 10 (*Figure 1(i,j)) are identical to Designs 3 and 4, with the exception that a cylindrical water bolus (*h* = 120 mm) was introduced to fill the air gap between the RF arrays and the human head for better coupling of the RF waves to the body [33].

A prerequisite of MRI is the perpendicularity of the magnetic transmission field (*B*_1_^+^) and the static magnetic field of the MR system (*B*_0_). Due to the radiation pattern of bow tie dipole antennae, the building blocks need to be arranged with their long axis parallel to the head-feet-direction. For Designs 2–10, copper shields were introduced between the RF building blocks to reduce nearest neighbor coupling. The position of each array was chosen so that the brain is centered within the *x*–*y*-plane (left–right, anterior–posterior) of the array and the TV for HTP is centered in the *z*-direction (superior–inferior). The combination of a voxel model with a specific RF applicator design will further be referred to as ‘configuration’.

All material parameters used for the EMF simulations are provided in Table 1.

**Table 1.** Materials and dielectric parameters used for electromagnetic field simulations.

Model	Component	Material	*σ* (S/m)	*ɛ*_r_	*ρ* (g/cm³)
Small tumor model	Tumor growth	Tumor	1.15	66.5	1025.5
	Duke	All tissues	IT’IS database		
	Closed layer of CSF	Dura → CSF	2.22	72.8	1007
Large tumor model	Tumor growth	Tumor	1.15	66.5	1025.5
	Brain	40% WM + 60% GM	0.52	50.3	1043
	Brainstem	40% WM + 60% GM	0.52	50.3	1043
	Tissue	Fat	0.07	11.7	911
		Muscle	0.77	58.2	1090.4
	Skull	Bone	0.08	13.5	1908
	Eye	Vitreous humor	1.52	69.0	1004.5
	Optical nerve	Nerve	0.42	37.0	1075
	Chiasm				
	Spinal cord				
	Vessels	Blood	1.32	65.7	1049.8
	Cavities	Air	0	1	1
	Pituitary gland	Hypothalamus	0.85	62.5	1053
	Hypothalamus				
Heavy water resonator (design 1)	Dielectric resonator	Heavy water (D_2_O)	0.02	81	1000
	Casing	Plexiglass (PMMA)	0.025	3.6	1180
	Antenna substrate	FR-4	0.025	4.3	1800
	Bow tie antenna	PEC			
High permittivity resonator (designs 2–10)	Dielectric resonator	Ceramic slurry	0.2	200	6020
	Casing	Plexiglass (PMMA)	0.025	3.6	1180
	Antenna substrate	FR-4	0.025	4.3	1800
	Bow tie antenna	PEC			
	Water bolus (designs 9 + 10)	Deionized water	0.016	81	1000

WM: white matter; GM: gray matter.

Parameters sources: tumor tissue [34]; body tissues [35]; D_2_O, deionized water, ceramic slurry: bench measurement based on an open-end coaxial probe setup [36,37]; PMMA, FR-4: material datasheets.

### Electromagnetic field simulations

EMF simulations were performed using Sim4Life V3.4 (ZurichMedTech, Zurich, Switzerland), with a broadband excitation at 297 ± 50 MHz and a simulation time of *t* = 120 ns. The resolution was limited to a maximum step size of 3 mm within the skull and 5 mm within the lower region of the voxel model. A much finer resolution of down to 0.5 mm was applied to resolve the triangular shape of the bow tie dipoles.

### Human voxel models

To address patients’ individuality, EMF simulations were performed for two voxel models of the human head:

1. Human voxel model ‘Duke’ of the virtual family [34] (IT’IS Foundation, Zürich, Switzerland) was modified to include an intracranial sphere (*d* = 4 cm, *σ*_Tumor_=1.15 S/m, *ε*_r,Tumor_=66.5 [34]) mimicking a small (<*λ*/2) tumor in the right parietal region of the brain with a TV of *V*_target_=33.5 ml. To ensure a closed layer of cerebrospinal fluid (CSF) to account for a potential RF shielding effect [37], the dura of the voxel model was assigned electromagnetic material properties of CSF (*σ* = 2.22 S/m) [35]. This model will be further referred to as ‘*small tumor model*’.
2. To mimic the clinical scenario, a realistic voxel model representing a patient with GBM encompassing a large TV (>*λ*/2, *V*_target_=500 ml) was generated from a computed tomography (CT) scan of a patient [39]. The resulting voxel data exhibits the same resolution as the planning CT and distinguishes up to 20 labeled tissues, which were assigned EM material properties of tissue [35]. A closed layer of external CSF was generated by upscaling the size of the brain by 5% while maintaining its shape and overwriting existing voxels assigned to muscle and skull. Assigning EM properties of CSF to the created envelope resulted in a closed layer with a thickness of 2–3 mm depending on the local mesh. This model will be further referred to as ‘*large tumor model*’.

### Data processing

**Figure 2.**
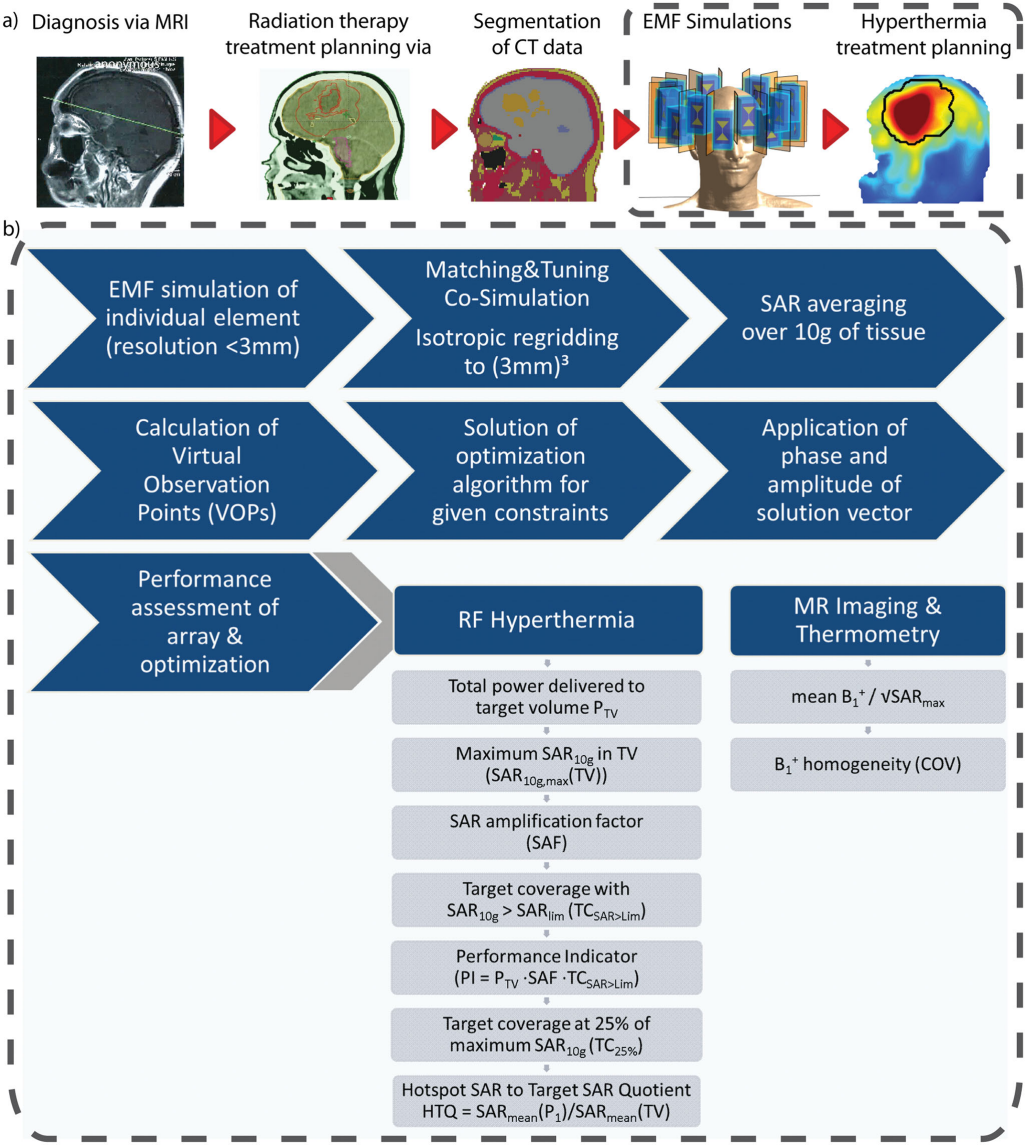
(a) Schematic presentation of the workflow from the diagnostic MRI of the patient to the SAR_10g_ distribution as the hyperthermia treatment planning. (b) Details of the simulation and optimization process from the tumor models to the metrics to be evaluated to assess the RF array for hyperthermia treatment planning and magnetic resonance imaging performance.

A circuit co-simulation was performed in MATLAB (MATLAB 2016b, The MathWorks, Natick, MA), optimizing the values of two lossy capacitances (equivalent series resistance = 0.15 Ω, equivalent series inductance = 1 nF) for channel-wise matching and tuning [40], minimizing the trace of the scattering matrix. The multi-channel point-SAR distributions were scaled based on the resulting scattering matrix and rebinned [41] to an isotropic grid of 3 mm. Our approach of thermal MR includes HTP and MR imaging. For the latter, guidelines ensuring patient safety rely on SAR averaging over cubes covering 10 g of tissue [42]. To be in compliance with these guidelines and to be consistent throughout our calculations, SAR_10g_ averaging [43] was also employed for the HTP. A good correlation of this averaging mass with temperature rise has been reported for the applied frequency range [43,44], in the head [45,46] and in hyperthermia treatment of a human subject [47]. This IEEE guideline foresees that averaging volumes comprising more than 10% air shall be assigned values from averaging cubes reaching into body instead of outwards. Since this does not generate new insights on the search for maximum SAR_10g_ values but rather assigns values to two locations, the locations resulting in a volume with a fill rate of less than 90% were discarded to decrease the data volume. Virtual observation points (VOPs) were calculated to accelerate the optimization [48,49], yielding a VOP-based result. The overestimation was chosen for each configuration so that the number of VOPs was 500 ± 10%. After optimization, the non-compressed field data were combined with the obtained phase and amplitude setting (full result). Values for local (SAR_10g,max_(healthy)) and global (*P*_Head_) exposure were compared with and without the use of VOPs and the amplitude of the excitation vectors was scaled to match the full results to the VOP-based results, thus partially removing the intrinsic overestimation by the VOP approach. A general overview of the workflow is given in Figure 2(a).

### Hyperthermia treatment planning

For the HTP, two optimization algorithms were developed, evaluated and compared for all RF coil array designs and for both tumor models. The goal of the optimization is to find excitation vectors defining the phase and amplitude setting for each channel. The resulting SAR pattern of the interfering incident electric fields causes the TV to heat up while the healthy tissue is spared of such exposure. We consider the scenario where multiple excitation settings can be played out consecutively so that their cumulative exposure constitutes the overall SAR pattern. The number of settings can be as high as the number of channels in the RF array. To suppress all solutions that contribute with less than 0.1% to the total delivered RF power, a threshold was introduced.

To describe the problem, we describe the power as the average power delivered in each of the *m* excitations, as previously described for a single excitation in [50]:

(1) $$P=\frac{1}{m}\sum_{j=1}^{m}x_{j}^{H}Qx_{j},$$

where *x_j_* are the individual excitation vectors and Q is the positive (semi)definite (psd) power correlation matrix of the tumor [51]. The dimension *N* of the vector corresponds to the number of channels of the system. With a local SAR matrix $S_{i}$ for each healthy voxel i, local SAR for these excitations equates to

(2) $$SAR_{i}=\frac{1}{m}\sum_{j=1}^{m}x_{j}^{H}S_{i}x_{j}.$$

A similar matrix can be constructed to calculate global SAR. Using appropriate compression methods, the SAR matrices can be replaced by a much smaller number of VOPs [48]. Both expressions for power deposition and local SAR are sums of quadratic forms, which can be rewritten using the trace operator to yield

(3) $$\begin{array}{c} tr(\frac{1}{m}\sum_{j=1}^{m}x_{j}^{H}Qx_{j})=\frac{1}{m}\sum_{j=1}^{m}tr(x_{j}^{H}Qx_{j})=\frac{1}{m}\sum_{j=1}^{m}tr(QX_{j})=\frac{1}{m}tr(Q\sum_{j=1}^{m}X_{j})\\ =\frac{1}{m}tr(\mathrm{QY}) \end{array}$$

By construction, Y is a positive (semi)definite matrix formed from the sum of outer products of the individual excitation vectors. We now seek to find

(4) $$\begin{array}{l} \mathrm{maximize}\;tr(\mathrm{QY})\\ \text{subject to }tr(S_{i}Y)\leq c_{i}\\ Y\geq 0\;(Y\;\text{is psd}) \end{array}$$

where $c_{i}$ are the SAR constraints to be observed.

This problem has a well-understood structure, appearing in the semidefinite relaxation approximation of quadratic form maximization [50], and can be solved via commonly available approaches such as the MATLAB-based modeling system for convex optimization (CVX [52]) using different constraints. The actual excitation vectors can be recovered from *Y* using its Eigen decomposition. The phase and amplitude settings are determined from the product of the Eigen vector and the square root of its Eigen value. Time multiplexing [53] is used when providing a better outcome, which is automatically determined by the algorithm.

First, the optimization goal was set to maximize total power absorption in the TV:

(5) $$\begin{array}{l} \mathrm{maximize}\text{ tr}(\mathrm{QY})\\ \text{subject to}\;tr(V_{i}Y)\leq c_{i}\\ tr(\mathrm{PY})\leq P_{\max} \end{array}$$

where *tr* is the trace of matrix; ***Y*** is the solution matrix (psd); ***Q*** is the tumor-SAR matrix; ***V_i_*** is the SAR matrices of the generalized VOPs; *P*_max_ is the power limit chosen for the optimization. Constraints to be defined are SAR_10g,max_ (healthy tissue), the total power delivered to the healthy part of the head *P*_Head_ (i.e., the remaining part of the head after numerically removing the TV so that the optimization does not limit itself) and the total available forward power *P*_Forward_. Head power deposition was constrained via the global SAR matrix ***P***. The possibility to limit *P_max_* is implemented in this approach but so far this has itself been the subject of investigations. *P_max_* was set so that SAR_Lim_ would be the limiting factor in all cases. For further considerations one has to bear in mind that *P*_Head_ is subject to varying absolute limits since the mass of the remaining exposed healthy tissue depends on patient anatomy and size of the TV. This optimization procedure will be further referred to as the ‘*power optimization*’.

The power optimization maximizes the total power delivered to the TV but does not consider the distribution of the power deposition inside the TV. Especially in large TVs, this could lead to a local SAR maximum while other regions of the TV experience low RF exposure. To address this shortcoming, a second optimization algorithm was implemented which homogenizes the power distribution within the TV by minimizing the deviation of every local SAR_10g_(TV) value from a given target SAR [50]. High target values favor the maximization of the power deposition while lower values favor its flatness:

(6) $$\begin{array}{l} \mathrm{minimize}\;s\\ \text{subject to}\;-s\leq tr(Q_{i}Y)-b\leq s\\ \begin{array}{c} tr(V_{i}Y)\leq c_{i}\\ tr(\mathrm{PY})\leq P_{\max} \end{array} \end{array}$$

where *s* is the optimization goal to minimize the deviation between each local SAR_10g_ value and *b*; *b*>0 is the targeted SAR_10g_(TV): to ensure that the algorithm targets voxels with low exposure that need increasing rather than voxels with high exposure that need reducing, we chose a high target SAR of *b* = 100 W/kg as previously reported in [54]; ***Y*** = solution matrix (psd); ***Q_i_*** = random subsample of the SAR matrices in the target region to reduce computational burden, considering that spatially dense sampling is not required. We used an undersampling factor of 0.5 resulting in about 20 matrices per cm³; ***V_i_*** = SAR matrices of the generalized VOPs and ***P*** = global SAR matrix. Constraints that can be defined are maximum SAR_10g_ in the healthy tissue SAR_10g,max_(healthy), the total power delivered to the healthy part of the head *P*_Head_ and the targeted SAR_10g_ in the TV b. This optimization procedure will be further referred to as the ‘*uniformity optimization*’.

To define a safe limit for SAR_10g,max_, we simplified the Pennes’ bioheat transfer equation [55], setting both the time-dependent and the conductive term to zero. Under the assumption of a basal brain perfusion of 50 ml/100 g/min, an average SAR of 50 W/kg results in a temperature increase of Δ*T* ≈+1.5 K without taking the protective response of thermoregulatory perfusion increase into account [12]. Based on this estimation, we chose the SAR_10g,max_(healthy)=SAR_lim_ = 40 W/kg for our optimizations.

For the small tumor, only the tumor volume was chosen as TV for optimization. For the large tumor model, the TV was defined along the clinical target volume (CTV) used for radiation therapy according to the current guidelines of the Radiation Therapy Oncology Group (RTOG, https://www.rtog.org), i.e., comprising the macroscopic tumor growth, an isotropic margin of 2 cm to cover potentially infiltrated brain tissue plus further edematous areas. All other tissues were considered healthy tissue and subject to the stringent constraint of a maximum allowed power deposition.

### HTP quality assessment

Quantitative assessment of SAR-based HTP quality offers a broad range of suggested metrics [56], each with their own merits and drawbacks, and is still of interest to define most promising surrogates for the resulting temperature distribution [57]. We assessed the following commonly [21,58,59] used metrics:

- *SAR amplification factor (SAF)*
  (7) $$\mathrm{SAF}={\mathrm{SAR}}_{10g,\mathrm{mean}}(\mathrm{TV})/{\mathrm{SAR}}_{10g,\mathrm{mean}}(\mathrm{healthy})$$
  which quantifies the ratio between the average power deposition in the TV vs. the healthy tissue but neglects local maxima and lacks information on the efficiency of treatment due to missing absolute power deposition levels
- *TC_25%_*
  The target coverage (TC) with 25% of the maximum SAR_10g_ (TC_25%_) is a measure for the SAR coverage of the TV while taking the exposure of the healthy tissue into account, but neglects the absolute SAR_10g_ values reached. It is quantified as the fraction of voxels in the TV exhibiting a local SAR_10g_ larger than 25% of the maximum exposure value found in the TV, SAR_10g,max_(TV).
- *Hotspot to target SAR quotient (HTQ)*
  (8) $$\mathrm{HTQ}=\;P_{1,\mathrm{mean}}({\mathrm{SAR}}_{10g}(\mathrm{healthy}))/{\mathrm{SAR}}_{10g,\mathrm{mean}}(\mathrm{TV})$$
  which focuses more on formation of local maxima than the SAF by comparing average SAR_10g_ values in the first percentile (P_1_) of healthy voxels exposed to the highest SAR_10g_ with the average SAR_10g_ in the TV, but again neglects the absolute values reached.
  In addition, to address some shortcomings of these metrics, we propose:
- *Performance indicator (PI)*
  (9) $$\mathrm{PI}\;(W/\mathrm{kg})={\mathrm{SAR}}_{10g,\max}(\mathrm{TV})\cdot \mathrm{SAF}\cdot {\mathrm{TC}}_{\mathrm{SAR}>\mathrm{Lim}}$$
  a combined measure addressing the shortcomings of the above metrics. While SAR_10g,max_(TV) reflects the absolute power deposition, SAF is a measure of how well healthy tissue is spared. Whether the exposure in the TV is focused to a small volume reaching high peak SAR_10g,max_(TV) values or exhibits a homogeneous power deposition in the TV is addressed by quantifying the fraction of voxels in the TV (TC) where exposure levels are higher than those allowed in the healthy tissue, TC_SAR>Lim_. We expect this last metric to be of major interest when comparing the two optimization algorithms.

A schematic overview of all metrics is summarized in Figure 2.

### MR imaging evaluation

To demonstrate that the RF applicators are also suitable for MRI and MRTh, magnetic transmission field (*B*_1_^+^) maps were calculated. A magnitude least square phase and amplitude *B*_1_^+^ shimming [60] was performed in MATLAB for an ellipsoidal region of interest covering the brain by using the dimensions of a bounding box around the brain to define the axes of the ellipsoid. The imaging performance of each design was evaluated based on the transmit efficiency $B_{1}^{+}/\sqrt{{\mathrm{SAR}}_{10g,\max}}$ and transmit uniformity across the ellipsoidal region of interest (coefficient of variation, COV).

## Results

### Radiofrequency antenna arrays

For both head models, the reflection coefficients for all configurations are below –32 dB. Among all designs, the coupling coefficients for Design 1 (SmallTumorModel) are the highest with –12.8 dB. For the building blocks with the high dielectric permittivity (*ɛ*_r_=200), all arrays could be perfectly matched and tuned to *S*_ii_<*S*_ij_<–17.8 dB. Such ceramic slurry can be produced using a mixture of BaTiO_3_ and CaTiO_3_ powder immersed in D_2_O [27]. The highest scattering parameters for all designs are given in Table 2 (full matrices are provided in the Supplementary Figure 1.2).

**Table 2.** Summary of all metrics assessing the performance of the RF-applicators, including the worst reflection and coupling coefficients (column 1, ‘Scattering’), the Hyperthermia Treatment Planning (HTP) results using the power optimization (column 2, ‘Power optimization’), the HTP results using the uniformity optimization (column 3, ‘Uniformity Optimization’) with # indicating the number of additive excitation phase and amplitude settings as well as the results of the B_1_^+^ shimming (column 4, ‘MRI’) for the small tumor model (top half) and the large tumor model (bottom half).

	Scattering		Power optimization									Uniformity optimization									MRI	
	*S*_ii,max_ (dB)	*S*_ij,max_ (dB)	#	SAR_10g,max_(TV) ($\frac{W}{\mathrm{kg}}$)	SAF	TC_SAR>Lim_ (%)	*P*_Head_ (W)	*P*_Tumor_ (W)	PI ($\frac{W}{\mathrm{kg}}$)	TC_25%_ (%)	HTQ	#	SAR_10g,max_(TV) ($\frac{W}{\mathrm{kg}}$)	SAF	TC_SAR>Lim_ (%)	*P*_Head_ (W)	*P*_Tumor_ (W)	PI ($\frac{W}{\mathrm{kg}}$)	TC_25%_ (%)	HTQ	B_1_^+^ ($\frac{\text{μT}}{\sqrt{kg\cdot W}}$)	COV (%)
																						
Small tumor model																						
Design 1	*–32.5*	*–12.5*	2	*46.7*	2.8	*36.0*	74.2	*1.5*	*47*	100	*1.00*	2	*43.8*	2.4	*18.9*	81.2	*1.4*	*20*	100	*1.04*	**0.25**	21.7
Design 2	–89.5	–30	1	51.4	*2.7*	47.2	86.2	1.6	66	100	0.90	2	48.5	*2.2*	57.4	104.2	1.6	62	100	0.92	0.21	19.2
Design 3	–71.5	–19.5	1	53.9	3.6	65.3	70.9	1.7	126	100	0.84	2	53.1	3.1	**77.2**	85.8	**1.8**	127	100	0.84	0.14	16.4
Design 4	–91	–30.5	1	56.3	3.4	73.3	77.0	**1.8**	140	100	0.79	2	54.8	3.0	72.9	86.1	**1.8**	120	100	**0.82**	0.13	17.4
Design 5	–97	–28.5	2	59.0	4.6	65.5	55.1	**1.8**	178	100	0.81	2	**57.4**	3.5	66.9	75.9	**1.8**	135	100	0.84	0.08	14.3
Design 6	**–110**	**–40.5**	2	**59.3**	3.0	66.7	64.6	1.7	152	100	0.83	2	50.4	3.3	59.9	72.1	1.6	99	100	0.88	0.14	17.5
Design 7	–66	–20	2	57.1	4.6	73.0	47.6	*1.5*	191	100	**0.77**	2	51.9	3.5	69.9	70.1	1.7	125	100	0.85	*0.02*	11.0
Design 8	–67.5	–17.5	1	51.3	4.1	55.4	51.3	1.6	116	100	0.88	2	46.4	2.6	43.8	82.5	1.5	52	100	0.97	0.04	**9.9**
Design 9	–77.5	–29.5	1	55.8	6.2	70.6	38.5	**1.8**	244	100	0.83	1	53.0	5.1	65.2	44.2	1.7	178	100	0.87	0.08	24.1
Design 10	–73	–27.5	1	56.6	**6.6**	**74.6**	36.6	**1.8**	**277**	100	0.82	1	53.6	**5.6**	63.5	40.1	1.7	**191**	100	0.88	0.04	25.3
Large tumor model																						
Design 1	*–62*	*–21.5*	2	*57.3*	*2.0*	*18.8*	84.1	*13.6*	*21*	100	*1.21*	2	*45.2*	*1.8*	*3.2*	87.5	12.2	*3*	100	*1.28*	**0.59**	28.1
Design 2	–85.5	–30.5	2	73.4	2.0	37.2	104.5	16.6	55	100	0.97	2	48.1	2.0	4.6	73.4	*11.6*	4	100	1.11	0.05	25.1
Design 3	–88.5	–32.5	2	96.4	2.7	45.5	87.0	18.3	117	93	0.86	2	82.4	2.4	47.5	97.2	18.0	93	100	0.92	0.05	20.1
Design 4	–88.5	–32	1	94.8	2.9	43.0	74.7	18.0	120	86	0.85	2	80.9	2.6	45.8	84.4	18.1	95	100	0.91	0.09	19.7
Design 5	**–109.5**	**–44**	2	100.3	3.0	**59.1**	92.8	**20.6**	174	98	**0.77**	2	75.5	2.6	**49.6**	95.7	18.0	99	100	0.88	0.10	20.4
Design 6	–107	–40.5	1	**108.3**	3.5	55.6	72.7	**20.6**	**210**	84	0.79	2	91.3	3.0	46.7	76.5	**18.7**	128	100	**0.85**	0.07	17.7
Design 7	–64.5	–20.5	2	70.7	2.4	47.9	91.8	17.8	82	100	0.87	2	59.6	2.5	31.9	84.7	16.0	47	100	0.98	0.05	**14.2**
Design 8	–65.5	–17.5	2	85.6	3.0	50.2	72.4	19.2	131	100	0.84	2	85.4	**3.5**	43.7	60.4	18.1	**131**	100	0.89	*0.04*	14.8
Design 9	–63.5	–24.5	1	104.6	3.1	32.5	62.4	16.3	105	*76*	0.99	2	**101.1**	3.0	29.7	63.6	15.9	89	*80*	1.02	0.07	28.6
Design 10	–71.5	–21	1	79.9	**3.8**	46.3	55.6	18.9	141	93	0.91	3	69.5	3.0	29.9	60.5	16.9	63	100	1.06	0.07	30.5

The best results are highlighted in **green and bold** while the worst results are *red and italic*.

### Small tumor model

**Figure 3.**
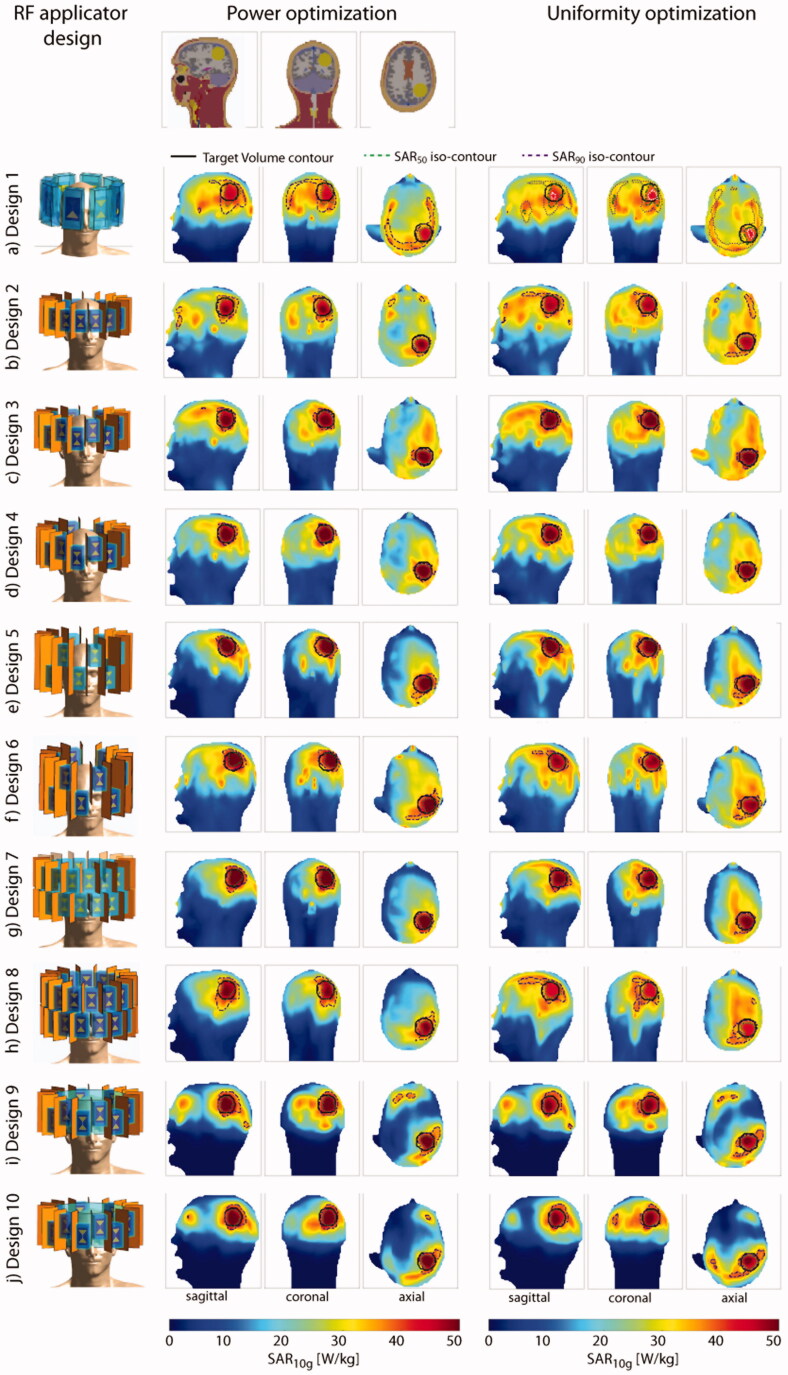
Top row: sagittal, coronal and axial section through the small tumor model. Left: description and front view of each design simulated together with the small tumor model; maximum intensity projection of the SAR_10g_ distribution after hyperthermia treatment planning for the power optimization (center) and the uniformity optimization (right).

**Figure 4.**
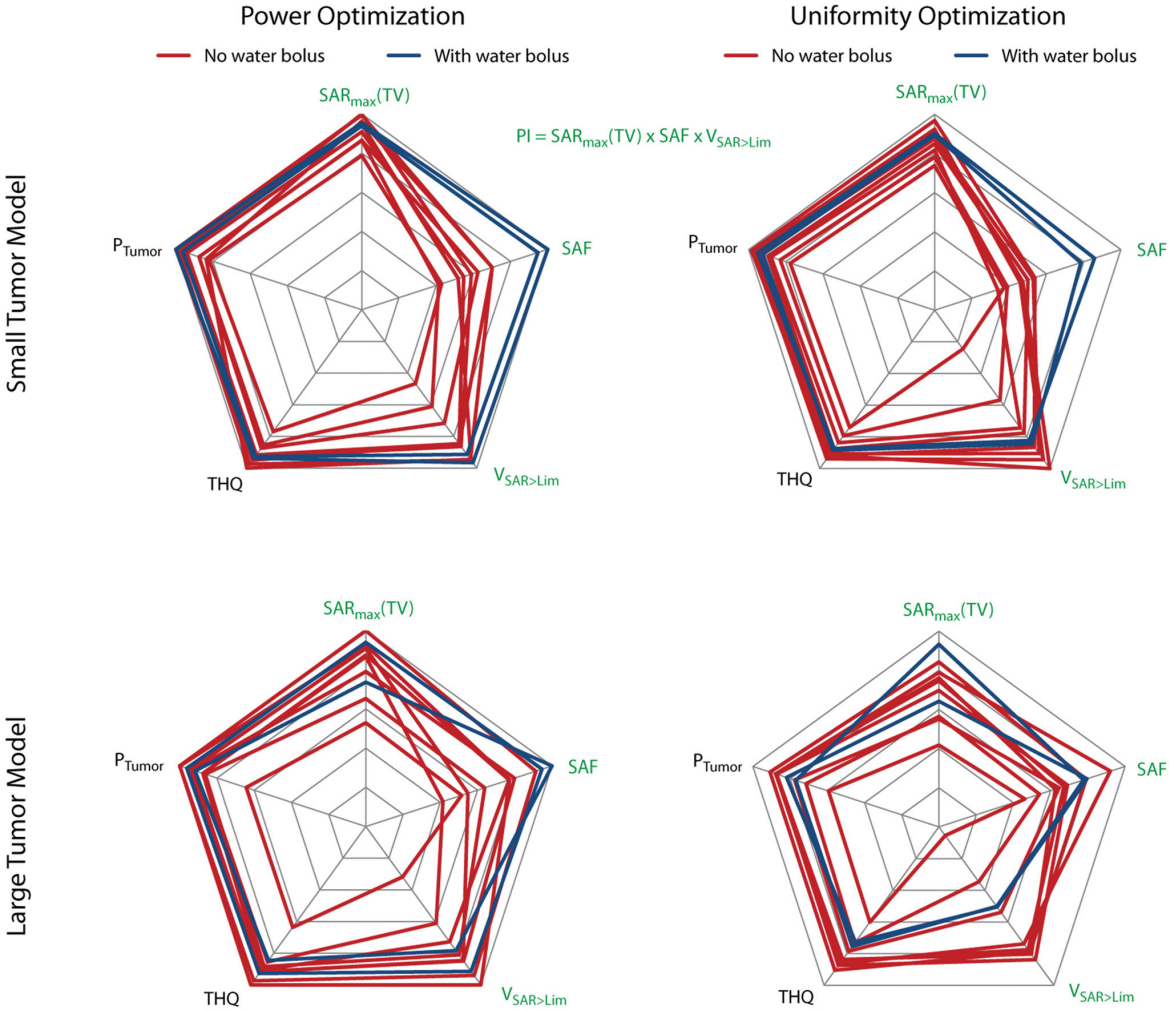
Qualitative comparison of the metrics used to assess HTP quality. The top row displays the results obtained for the small tumor model; the bottom row shows the results for the large tumor model. The results in the left column are obtained using the power optimization; the results of the uniformity optimization are in the right column. Blue lines highlight designs 9 and 10 equipped with a water bolus. All values are normalized to their respective intra-design, intra-optimizer maximum. Metrics labeled with a green font combine to the performance indicator. For better visualization so that higher values are always better, we plotted the reciprocal of the HTQ (1/HTQ = THQ). For the small tumor model, adding the water bolus clearly improved the SAF and pushed toward the highest values for SAR_max_(TV) and *V*_SAR>Lim_. For the large tumor model, the results are very heterogeneous; the water bolus does not add a clear improvement.

The *power optimization* algorithm yielded an increased RF power deposition with a TC_25%_ of 100% in the small TV for all configurations. For Designs 1, 5, 6 and 7, the cumulative exposure of two excitation settings (phase and amplitude) yields the optimized SAR_10g_ distribution. The solution for all other designs consists of one excitation vector. The maximum intensity projections (MIPs) for the obtained SAR_10g_ distributions overlaid with the SAR_50_ and SAR_90_ iso-contours are shown in the center column of Figure 3. Additionally, the SAR_10g_ maps in three orthogonal slices positioned in the center of the TV are shown in the center column of Supplementary Figure 3.2. The metrics are visualized in the top left of Figure 4 while all values are listed in Table 2.

Increasing the number of channels from 8 to 16 when moving from Design 1 to Design 2 showed an increase in RF power deposition in the TV (*P*_Tumor_(Design 2)=1.6 W vs. *P*_Tumor_(Design 1)=1.5 W) – but also in the healthy part of the brain. Moving to Design 3 and introducing the third focusing dimension significantly decreased the exposure of the healthy tissue (SAR_mean_(healthy, Design 3)=11.8 W/kg vs. SAR_mean_(healthy, Design 2)=14.6 W/kg). Arranging the antennae in the elliptical manner (Design 4) allowed for higher total incident power (*P*_Head_(Design 4)=77.0 W vs. *P*_Head_ (Design 3)=70.9 W) before reaching the local SAR_10g_ (healthy) limit, yielding a performance indicator of PI = 88 W/kg, according to Equation (9). The HTQ as defined in Equation (8) decreases from HTQ (Design 1)=1.00 to HTQ (Design 4)=0.79 with these design improvements. Increasing the longitudinal coverage of the head (Design 5) enabled further focusing of the RF waves to the TV (PI = 178 W/kg). Changing to the elliptical setup of Design 6 decreased the PI (PI(Design 6) = 152 W/kg). Adding more degrees of freedom by increasing the number of channels to 32 further increased the PI for the circular but not for the elliptical arrangement (PI(Design 7)=191 W/kg, PI(Design 8) = 116 W/kg). Again, the HTQ values support these findings. The introduction of the water bolus allowed for a significant reduction of surface SAR_10g_. As a result, Designs 9 and 10 are the only designs where the limiting SAR_10,max_ = 40 W/kg was reached adjacent to the TV rather than at the surface. In combination with a significant decrease in exposure of healthy tissue (*P*_Head_(Design 9) = 38.5W vs. *P*_Head_(Design 3) = 70.9 W and *P*_Head_(Design 10) = 36.6 W vs. *P*_Head_(Design 4) = 77.0 W), this resulted in significantly better performance for both arrays with water bolus (PI(Design 9) = 244 W/kg, PI(Design 10)= 277 W/kg). The HTQ values are among the lowest (HTQ(Design 9) = 0.83, HTQ(Design 10)=0.82) but outperformed by Design 7. Overall, the differences in HTQ for all 10 designs are very low, except for Design 1, showing the highest HTQ (HTQ(Design 1)=1.00).

The *uniformity optimization* yielded a superposition of two excitation phase and amplitude settings for all arrays without a water bolus (Designs 1–8), where one setting was found sufficient. The MIPs for the obtained SAR_10g_ distributions overlaid with the SAR_50_ and SAR_90_ iso-contours are shown in the right column of Figure 3. Additionally, the SAR_10g_ maps in three orthogonal slices positioned in the center of the TV are shown in the right column of Supplementary Figure 3.2. The metrics are visualized in the top right of Figure 4 while all values are listed in Table 2. An increased TC_SAR>Lim_ of the TV was achieved for Designs 2, 3 and 5 when compared to the power optimization. However, in all cases, the improvement comes at the expense of a decrease in SAF, as defined in Equation (7), and maximum SAR_10g_ in the TV. The TC_25%_ of 100% could be maintained for all configurations. The HTQ values show the same behavior throughout the design iterations but exhibit a minor increase overall (lowest: HTQ(Design 4)=0.82, highest: HTQ(Design 1)=1.04). As a consequence, employing the uniformity optimization with the small tumor model showed no significant PI improvement.

### Large tumor volume

**Figure 5.**
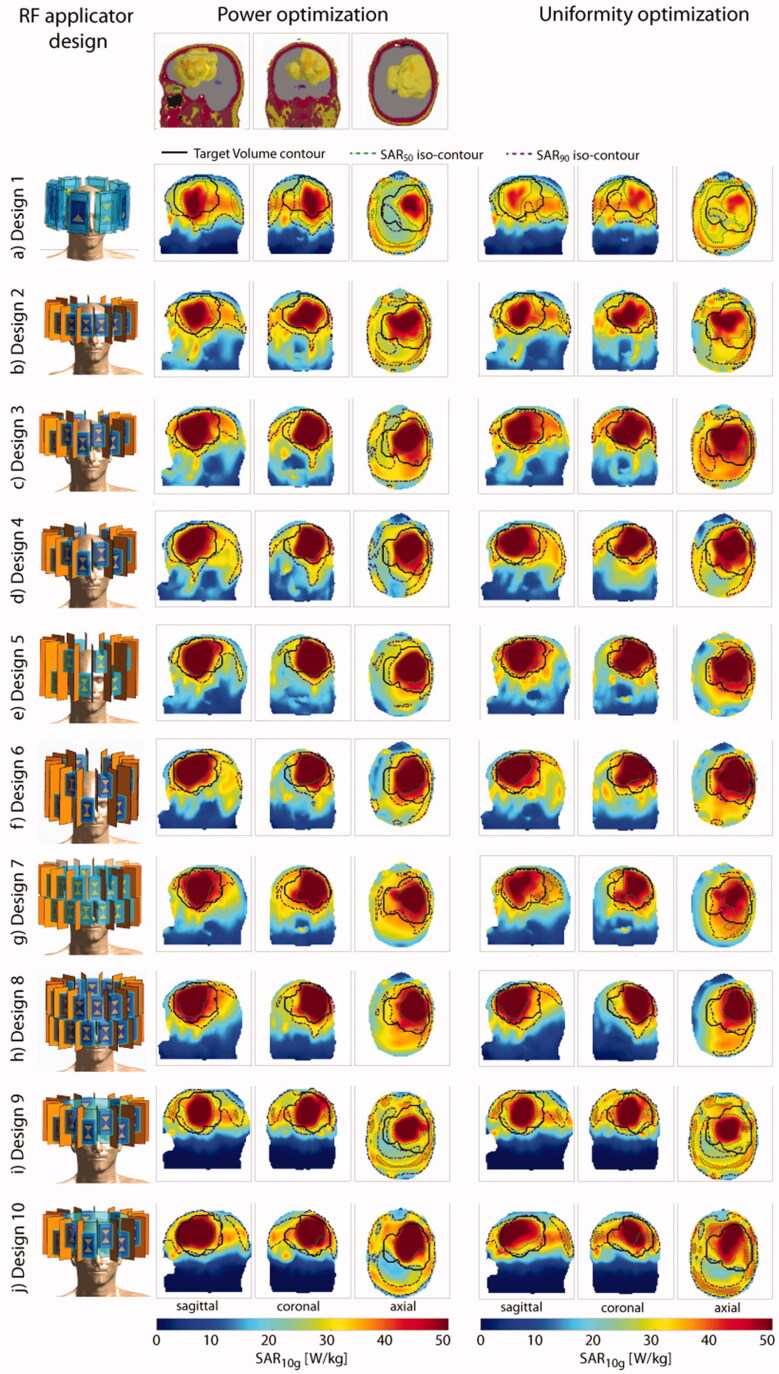
Top row: sagittal, coronal and axial section through the large tumor model. Left: description and front view of each design simulated together with the large tumor model; maximum intensity projection of the SAR_10g_ distribution after hyperthermia treatment planning for the power optimization (center) and the uniformity optimization (right).

The more frontal location of the large TV (*V* = 500 ml) resulted in a higher power deposition in the entire brain and including the region of the eyes and the nose. The MIPs for the obtained SAR_10g_ distributions overlaid with the SAR_50_ and SAR_90_ iso-contours are shown in Figure 5. Additionally, the SAR_10g_ maps in three orthogonal slices positioned in the center of the TV are shown in the Supplementary Figure 5.2. The metrics are visualized in the bottom row of Figure 4 while all values are listed in Table 2.

The *power optimization* yielded a superposition of two excitation phase and amplitude settings for Designs 1, 2, 3, 7 and 8. The TC_25%_ was well above 75% for all configurations (84–100%) without the water bolus. Significant differences were observed for TV coverage by using a higher amount of channels. At the same time, a reduction in exposure of healthy tissue was achieved. The PI increased with every iteration of the antenna array up to Design 6 (PI_min_=21 W/kg, PI_max_=210 W/kg), which also exhibits the best HTQ value (HTQ(Design 6)=0.79). This level of exposure could not be maintained when moving to Design 7, due to the high density of transmit elements close to the nose, resulting in the formation of a local SAR maximum (PI(Design 7)=82 W/kg, HTQ = 0.87). This could partly be countered with the elliptical arrangement in Design 8 (PI(Design 8)=131 W/kg, HTQ(Design 8)=0.84). The inclusion of a water bolus in Designs 9 and 10 did not show the same improvement as for the small TV. While an increase in SAR_max_(TV) and SAF could be observed, the enhanced RF focusing led to a significant decrease in TC_SAR>Lim_ and TC_25%_ (PI(Design 9)=105 W/kg vs. PI(Design 3)=117 W/kg, HTQ(Design(9)=0.99 vs. HTQ(Design 3)=0.86, TC_25%_(Design 9)=76% vs. TC_25%_(Design 3)=93%). Improvements were observed by moving to the elliptical Design 10 (PI(Design 10)=141 W/kg, HTQ(Design(10)=0.91, TC_25%_(Design 10)=93%), but the results did not match Design 6.

The *uniformity optimization* was designed to improve the HTP for large TVs by spreading the power deposition in the TV. This was achieved by a superposition of two excitation settings for all arrays but Design 10, where a third setting contributed another 8% to the total delivered power. In all non-bolus designs, the second phase setting contributed as much as 16–40% to the total delivered power vs. 0–31% in the power optimization.

The modest performance achieved with the in-plane arrays of Designs 1 and 2 was substantially enhanced when increasing the longitudinal coverage (PI(Design 5)=99 W/kg, PI(Design 3)=93 W/kg, PI(Design 2)=26 W/kg; HTQ(Design 5)=0.88, HTQ(Design 3)=0.92, HTQ(Design 2)=1.28). The additional degree of freedom of 32 channels in Design 7 did not add to the improvement but rather boosted the formation of a local SAR maximum (PI(Design 7)=47 W/kg, HTQ(Design 7)=0.98). All elliptical configurations outperformed the circular counterparts in PI (PI(Design 4)=95 W/kg vs. PI(Design 3)=93, HTQ(Design 4)=0.91 vs. HTQ(Design 3)=0.92; PI(Design 6)=128 W/kg vs. PI(Design 5)=99 W/kg, HTQ(Design 6)=0.85 vs. HTQ(Design 5)=0.88; PI(Design 8)=131 W/kg vs. PI(Design 7)=47 W/kg, HTQ(Design 8)=0.89 vs. HTQ(Design 7)=0.98). Minor improvements in SAF and as a result in PI were found when increasing the number of channels from 16 to 32 in the elliptical arrangement (PI(Design 8)=131 W/kg vs. PI(Design 6)=128 W/kg). Adding the water bolus significantly decreased the PI results for Design 9 (PI(Design 9)=63 W/kg vs. PI(Design 3)=17 W/kg) due to overfocusing (TC_SAR>Lim_(Design 9)=29.7% vs. TC_SAR>Lim_(Design 3)=47.5%), despite an increase in maximum power deposition (SAR_max_(TV,Design 9)=101.1 W/kg vs. SAR_max_(TV,Design 3)=82.43 W/kg) with poorer results for HTQ and TC_25%_ (HTQ(Design 9)=1.02 vs. HTQ(Design 3)=0.92, TC_25%_(Design 9 = 80% vs. TC_25%_(Design 3)=100%). For Design 10, no improvement could be found (PI(Design 10)=63 W/kg, HTQ(Design 10)=1.06, TC_25%_(Design 10)=100%). Using the uniformity optimization helped TC_25%_ values reach 100% for all arrays except Design 9, where only 80% could be reached, stating a significant improvement over the power optimization.

### MR imaging evaluation

For the small tumor model, the best B_1_^+^ transmit efficiency was obtained for Design 1 ($B_{1}^{+}/\sqrt{{\mathrm{SAR}}_{10g,\max}}(\mathrm{Design}\;1)=0.25\mu T/\sqrt{W\cdot \mathrm{kg}}\pm 21.7\%$), which was comparable to Design 2$(B_{1}^{+}/\sqrt{{\mathrm{SAR}}_{10g,\max}}(\mathrm{Design}\;2)=0.21\mu T/\sqrt{W\cdot \mathrm{kg}}\pm 19.2\%$). The transmit efficiency was lowest for Design 7 $(B_{1}^{+}/\sqrt{{\mathrm{SAR}}_{10g,\max}}(\mathrm{Design}\;7)=0.02\mu T/\sqrt{W\cdot \mathrm{kg}}$) although the homogeneity improved (COV(Design 7)=11.0%).

For the large tumor model, all designs except Design 1 $(B_{1}^{+}/\sqrt{{\mathrm{SAR}}_{10g,\max}}(\mathrm{Design}\;1)=0.59\mu T/\sqrt{W\cdot \mathrm{kg}}\pm 28.1\%$) showed lower or similar B_1_^+^ transmit efficiencies compared to the small tumor model. Design 8 showed the lowest B_1_^+^ transmit efficiency ($B_{1}^{+}/\sqrt{{\mathrm{SAR}}_{10g,\max}}(\mathrm{Design}\;8)=0.04\mu T/\sqrt{W\cdot \mathrm{kg}},$ COV(Design 8)=14.8%). A synopsis of the transmit efficiency and transmit uniformity is provided in the right column of Table 2.

## Discussion

This work examined the applicability of high density annular-phased-array RF applicators and HTP in a small (<*λ*/2, *V*_target_=33.5 ml) and large (>*λ*/2, *V*_target_=500 ml) TV in the brain using EMF simulations.

Regarding the metrics used to examine our HTP quality, we found TC_25%_ to be least informative, reaching 100% in 33 out of 40 HTP results, even in cases where SAR_10g_ distribution did not appear promising for treatment. The validity of our proposed PI is supported by its correlation with the well-established HTQ. We believe that the PI adds to the HTP quality assessment since it offers a measure for absolute SAR and a higher differentiation compared to the HTQ. This being said, this work is, to the best of our knowledge, the first to present an HTQ <1 for HTP in the head [61].

For the small tumor model, the power optimization clearly outperformed the uniformity optimization. The TV diameter of 4 cm approximates a third of the wavelength in tissue at 297 MHz and thus stresses the physical limits of RF focusing, thereby leading to good TC. Adding the uniformity as an optimization criterion limits maximization of delivered RF power. In conclusion, HTP for small TVs should be preferably performed using the power optimization. When lifting the SAR_10g,max_ constraint of healthy tissue, this optimization converges to an optimization of the SAF [21].

An incremental improvement in HTP performance was accomplished when moving from Design 1 to Design 5. Enabling the longitudinal steering in Design 3 resulted in increased sparing of healthy tissue. The elliptical arrangement of Design 4 afforded enhanced control over superficial power deposition. Without the use of a water bolus, the best RF focusing was achieved by using the highest degree of freedom for the 32 elements in Design 7. Design 8 could not maintain this performance due to higher power deposition in the temporal regions of the head.

Employing the uniformity optimization for the large TV resulted in a higher coverage TC_SAR>Lim_ for only Designs 3 and 4. Further expenses at the cost of SAR_max_ and SAF do not justify the use of the uniformity optimization, even though its principal effectiveness was shown by the higher contribution of secondary excitation settings and higher TC_25%_ values.

Given the large geometrical extent of the TV in the large tumor model, the increased longitudinal coverage was essential to obtain good HTP results, peaking in the results found for the power optimization for Design 6. All elliptical arrangements allowed for better results than their circular counterparts. Increasing the number of elements from 16 to 32 added no clear improvement. Only TC_25%_(Design 8) in the power optimization and SAF and TC_SAR>Lim_(Design 8) in the uniformity optimization could be improved. All other metrics show no improvement, even a poorer performance, upon increasing the channel count.

Adding the water bolus supported an improvement in coupling of the RF energy to the body as well as focusing for the small tumor model. For the large tumor model, the increased focusing lead to a decreased TV coverage in Design 9. Moving to the elliptical Design 10 counters for some of this behavior but does not outperform the arrays without a water bolus. These designs show encouraging HTP results, whereby high enough power deposition in the TV may be reached without the requirement of a water bolus as coupling medium. This would substantially benefit patient comfort.

From an engineering perspective, our results suggest that it is possible to limit the RF applicator to 16 building blocks for both tumor models. This simplifies the RF applicator setup. Such a setup would be compatible with state-of-the-art 7.0 T MR instruments offering up to 16 RF amplifiers (*P_max_*=2 kW) for parallel transmission. In addition, the low coupling coefficients suggest that the copper shields might become obsolete, which would relax engineering and manufacturing constraints.

To summarize, our results revealed that the 16 element interleaved array using an elliptical arrangement provided the best results for the small tumor model, while the 16 element elliptical two row arrangement showed the best results for the large tumor model. This supports the idea of a ‘sliding applicator’, where the two rows of RF antennae can be displaced with respect to each other, adapting the longitudinal coverage of the brain individually in response to the size of the TV. Since our findings support the use of a water bolus for the small TV but not for the large TV, the question as to whether the engineering effort of designing an adaptable water bolus for such an applicator is weighed by the benefit will need to be answered by the ongoing investigation into a higher number of realistic tumor models. The elimination of the copper shields would ease the realization of this applicator.

To conclude, this work adds to the literature by examining integrated RF applicator concepts for thermal interventions in the brain including treatment planning based on realistic patient models. Swift translation of the RF applicator designs examined with numerical simulations into experimental prototypes remains conceptually appealing and an ambitious undertaking en route to clinical feasibility studies of thermal interventions of GBM.

## Supplementary Material

Fig5-2__LargeTumor_centralAxis

Fig3-2__SmallTumor_centralAxis

Fig1-2__S-Matrices
