# Supplementary figures and images for: Radiofrequency applicator concepts for thermal magnetic resonance of brain tumors at 297 MHz (7.0 Tesla)
Source: Int J Hyperthermia. 2020 Jun 2;37(1):549–63. doi: 10.1080/02656736.2020.1761462 (PMC8352381; doi:10.1080/02656736.2020.1761462)

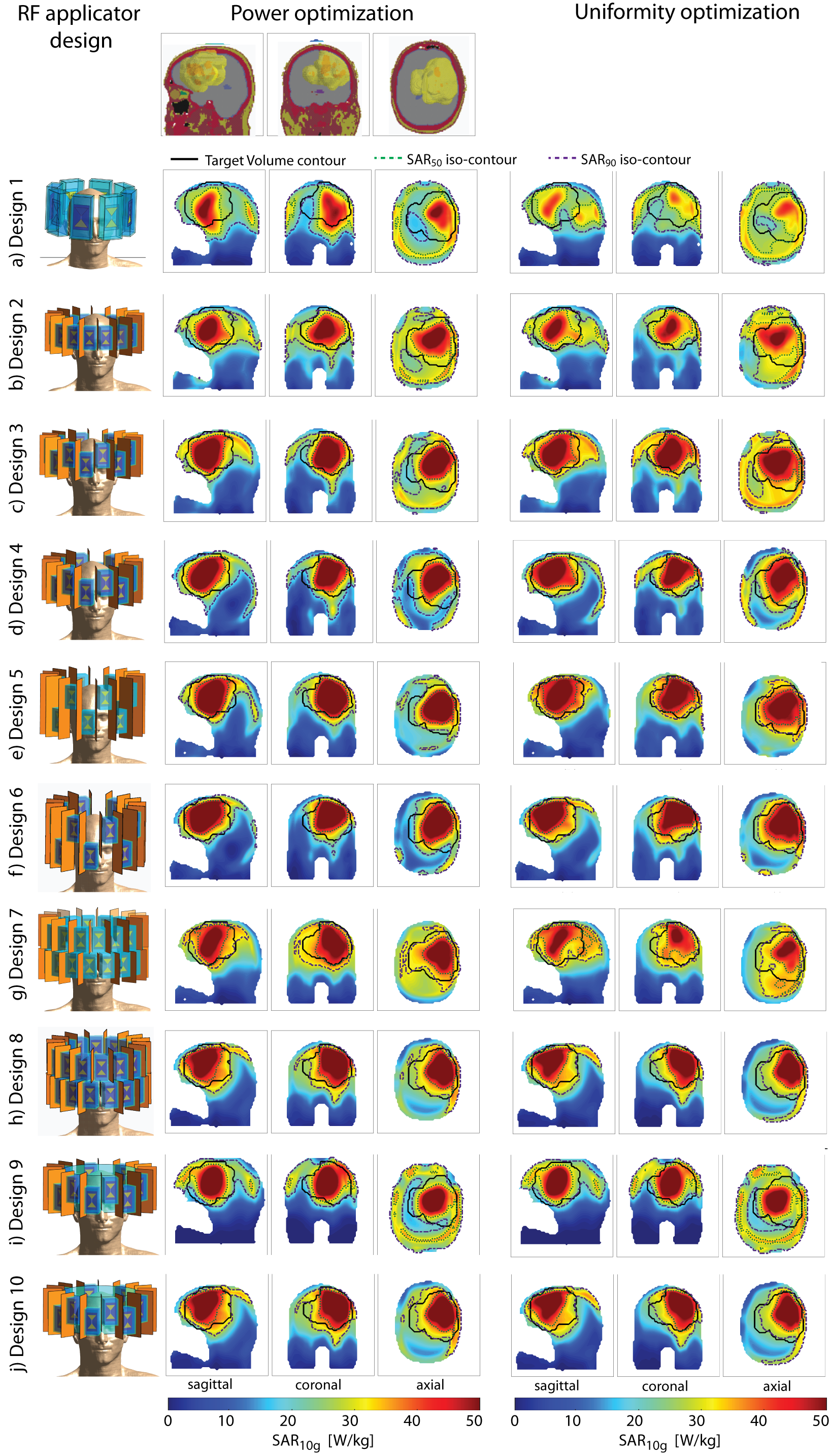

Supplement: Fig5-2__LargeTumor_centralAxis [file IHYT_A_1761462_SM7856.tif]

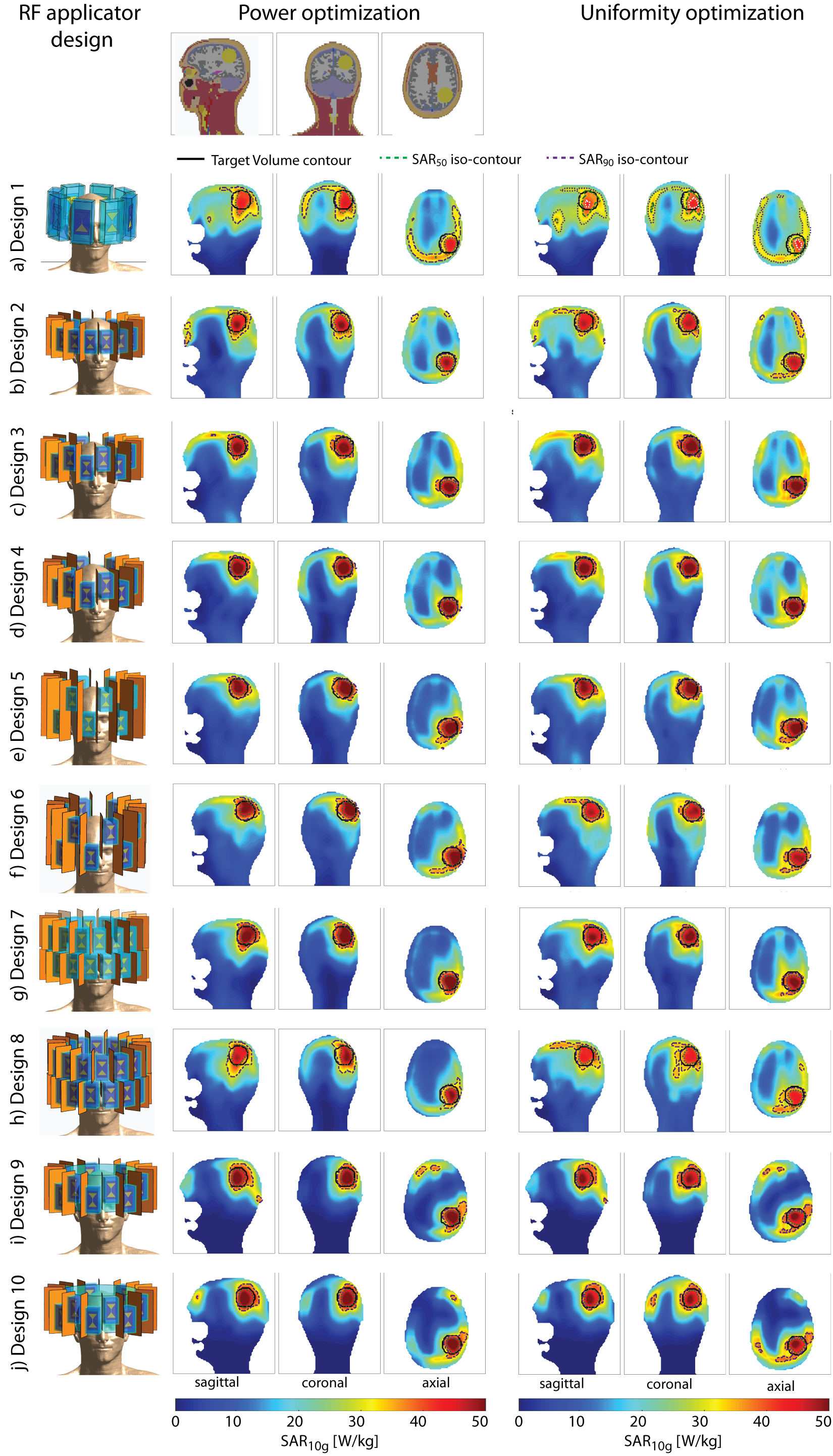

Supplement: Fig3-2__SmallTumor_centralAxis [file IHYT_A_1761462_SM7845.tif]

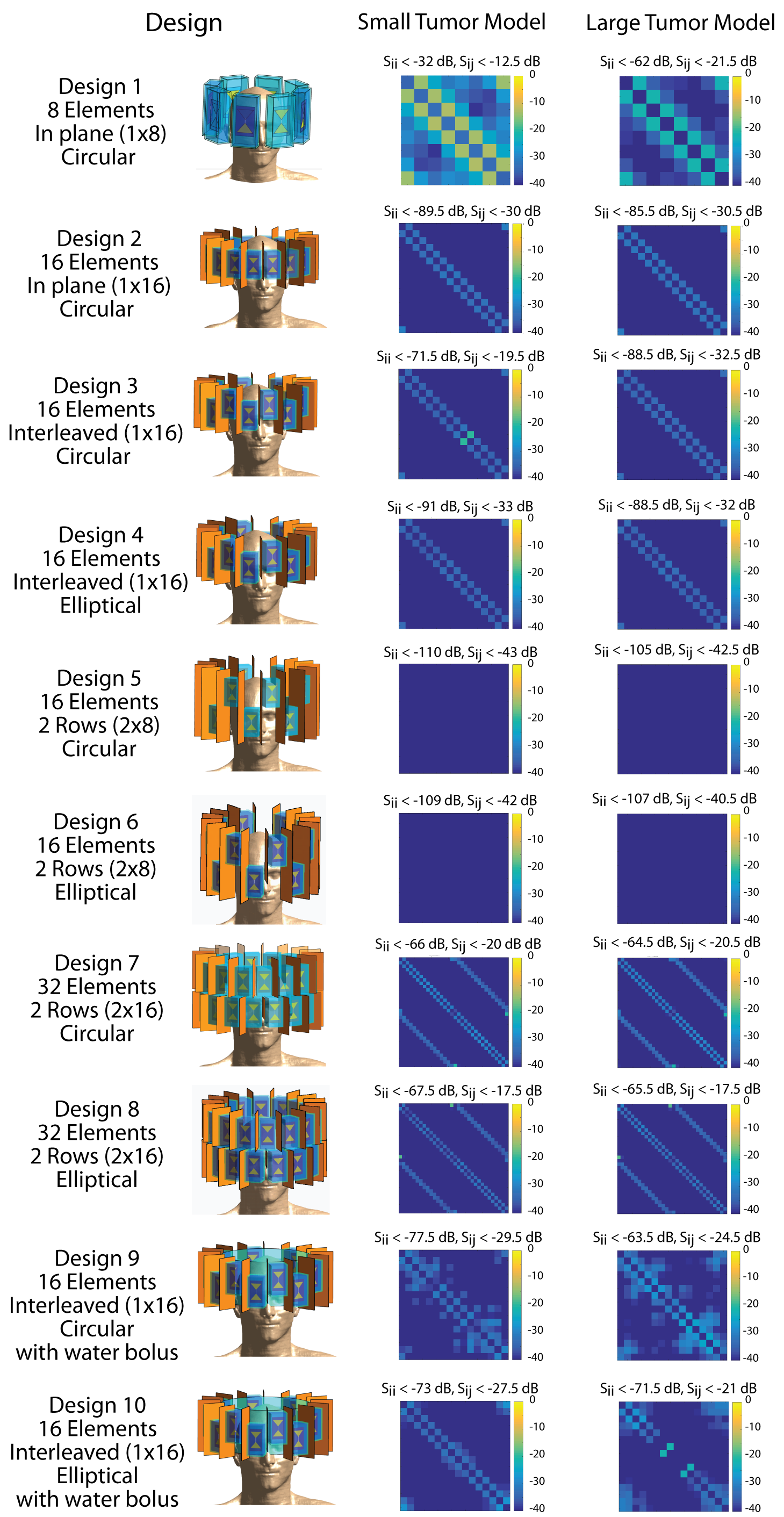

Supplement: Fig1-2__S-Matrices [file IHYT_A_1761462_SM7819.tif]
